# Prevalence and Determinants of COVID-19 Vaccination Uptake Were Different between Chinese Diabetic Inpatients with and without Chronic Complications: A Cross-Sectional Survey

**DOI:** 10.3390/vaccines10070994

**Published:** 2022-06-22

**Authors:** Junjie Xu, Siyu Chen, Ying Wang, Lingrui Duan, Jing Li, Ying Shan, Xinquan Lan, Moxin Song, Jianzhou Yang, Zixin Wang

**Affiliations:** 1 Clinical Research Academy, Peking University Shenzhen Hospital, Peking University, No. 1120, Lianhua Road, Futian District, Shenzhen 518036, China; xjjcmu@163.com (J.X.); 18211020063@fudan.edu.cn (J.L.); sylvia.shanboo@gmail.com (Y.S.); 2 Jockey Club School of Public Health and Primary Care, Faculty of Medicine, The Chinese University of Hong Kong, Hong Kong 666888, China; chensiyu@link.cuhk.edu.hk; 3 School of Epidemiology and Public Health, Shanxi Medical University, Taiyuan 032000, China; wangying@b.sxmu.edu.cn (Y.W.); dlr0875@b.sxmu.edu.cn (L.D.); 4 Department of Epidemiology, China Medical University, No. 77, Puhe Road, Shenyang North District, Shenyang 110122, China; xqlan@cmu.edu.cn (X.L.); mxsong@cmu.edu.cn (M.S.); 5 Department of Public Health and Preventive Medicine, Changzhi Medical College, Changzhi 046000, China

**Keywords:** diabetes, complications, coronavirus disease 2019 (COVID-19) vaccination uptake, health belief model, China

## Abstract

The health of people with chronic diabetes mellitus (DM) complications will worsen following coronavirus disease 2019 (COVID-19) infection. This cross-sectional study compared perceptions and factors related to COVID-19 vaccination uptake between subgroups of DM inpatients with and without chronic complications in China. A multivariate logistic regression model was used for data analysis. Of the 645 participants, those without any complications reported significantly higher uptake of at least one dose of COVID-19 vaccination (43.2% versus 11.2%, *p* < 0.001). For people with chronic DM complications, a perception of higher risk and severer consequences of COVID-19 infection, a belief that doctors would suggest they receive COVID-19 vaccination, and a belief that relatives’ vaccination uptake would influence their own decision to receive a COVID-19 vaccination were all associated with higher COVID-19 vaccination uptake. For their counterparts without chronic complications, a perception of severer consequences of COVID-19 infection, a belief that receiving COVID-19 vaccination could reduce the risk of infection, and a belief that relatives’ vaccination uptake would influence their own decision to receive a COVID-19 vaccination were all associated with higher COVID-19 vaccination uptake. Concerns about the safety and the side effects of vaccination were negatively associated with COVID-19 vaccination uptake in both groups of DM patients. Different strategies might be applied to promote COVID-19 vaccination uptake in DM patients with and without chronic complications.

## 1. Introduction

Globally, diabetes mellitus (DM) affects approximately 537 million people [1]. China has the largest number of people with DM (140 million), accounting for 11% of its entire population [1,2]. The coronavirus disease 2019 (COVID-19) pandemic is a serious health threat worldwide [3]. People with DM are at higher risk of morbidity and mortality from COVID-19 [4]. Possible causes include DM-related immunological dysfunction and increased inflammation [5], as well as increased adherence of COVID-19 to target cells [6]. In addition, COVID-19 infections have been associated with an increased risk of developing renal disturbances [7], neurologic injury [8], and other skin damage [9] among people with DM. Therefore, people with DM are considered a priority group to receive a COVID-19 vaccination. International health authorities consistently recommend COVID-19 vaccination for all people with DM [10,11,12,13]. In China, people with DM without acute complications (e.g., ketoacidosis, hyperosmolar states, lactic acidosis) are encouraged to receive a COVID-19 vaccination [14].

DM can affect many different organ systems and lead to serious chronic complications over time. Chronic DM complications include microvascular complications (neuropathy, nephropathy, and eye damage) and macrovascular complications (cardiovascular and cerebrovascular diseases, stroke, and peripheral vascular diseases) [9]. In China, more than half of people with DM have at least one chronic DM complication, which causes a substantial burden on health and the economy. The most common chronic DM complications include cardiovascular diseases (30.1%), cerebrovascular diseases (6.8%), neuropathy (17.8%), nephropathy (10.7%), eye damage (14.8%), and foot damage (0.8%) [2,15,16]. People with chronic DM complications are more vulnerable to COVID-19. Previous studies have shown that people with chronic DM complications in the past 5 years had an increased risk of mortality and intensive care unit (ICU) admission with longer hospital stays following COVID-19 infection compared to people with DM without such complications [17,18]. No report has compared the efficacy and side effects of COVID-19 vaccination between people with DM with and without chronic complications. The available evidence suggests that COVID-19 vaccination is beneficial for all people with DM [19].

Few published studies have investigated COVID-19 vaccine hesitancy among people with DM. The prevalence of vaccine hesitancy ranged from 14.2% in Italy [20] to 29% in Saudi Arabia [4], 24.7% in Malaysia [21], and 56.4% in China [22]. To the best of our knowledge, three studies have investigated actual uptake of COVID-19 vaccination among people with DM. The prevalence of uptake was 21.5% in India [23], 25.2%% in China [24], and 84.8% in Saudi Arabia [25]. The main reasons for vaccine hesitancy among people with DM included concerns about vaccine safety and side effects, perceived consequences of COVID-19 infection, relatives’ vaccination status, and suggestions made by physicians [4,20,21,22,23,24,25]. It is possible that people with DM who have chronic complications have different perceptions related to COVID-19 compared to their counterparts without such complications. People with chronic DM complications may perceive a higher risk and severer consequences of COVID-19. There is inadequate evidence about vaccine efficacy and safety for people with chronic DM complications. Therefore, people with chronic DM complications might have more concerns about these matters due to their poorer health conditions. Such differences in perception may lead to different levels of COVID-19 vaccination uptake among people with and without chronic DM complications. No study has tested such a hypothesis. Currently, the same information and health promotions related to COVID-19 vaccination are provided to people with DM regardless of the presence or absence of chronic complications. It is necessary to understand whether factors associated with COVID-19 vaccination uptake are different among people with and without chronic DM complications. If the associated factors are different between these two groups, future programs should consider using different intervention strategies for each group to better cater to their needs. According to the principle of social marketing, careful segmentation will improve the effectiveness of health promotion campaigns [26]. 

This study used the Health Belief Model (HBM) as a theoretical framework [27]. Previous studies have used the HBM to investigate COVID-19 vaccine hesitancy in different Chinese populations, including people with DM [22]. 

To address the knowledge gaps, this study compared COVID-19 vaccination uptake and levels of perceptions related to COVID-19 vaccination between subgroups of DM inpatients with and without chronic complications. This study also investigated factors associated with COVID-19 vaccination uptake in each subgroup. We hypothesized that there would be significant between-group differences affecting COVID-19 vaccination uptake and levels of perceptions related to COVID-19/COVID-19 vaccination. The associated factors of COVID-19 vaccination uptake would also be different between participants with and without chronic DM complications.

## 2. Materials and Methods

### 2.1. Study Design

This study was a cross-sectional study conducted among inpatients diagnosed with DM which was carried out at the endocrinology departments of Heping and Heji Hospitals, Changzhi Medical College (Changzhi, Shanxi, China) between April and August 2021. This study was a continuation of our published studies [22,24]. 

### 2.2. Participants

Inclusion criteria applied to patients (1) diagnosed with type 1 or type 2 DM who were hospitalized in the two participating hospitals throughout the study period, (2) aged at least 18 years, and (3) willing to provide written informed consent to complete the survey. Exclusion criteria applied to patients (1) with a diagnosis of mental illness or who had taken medication for a mental illness in the previous 3 months, or (2) who had apparent dementia symptoms or were unable to communicate verbally with the investigators. Physicians confirmed the first two inclusion and exclusion criteria based on their medical records.

### 2.3. Recruitment and Data Collection

**Figure 1:**
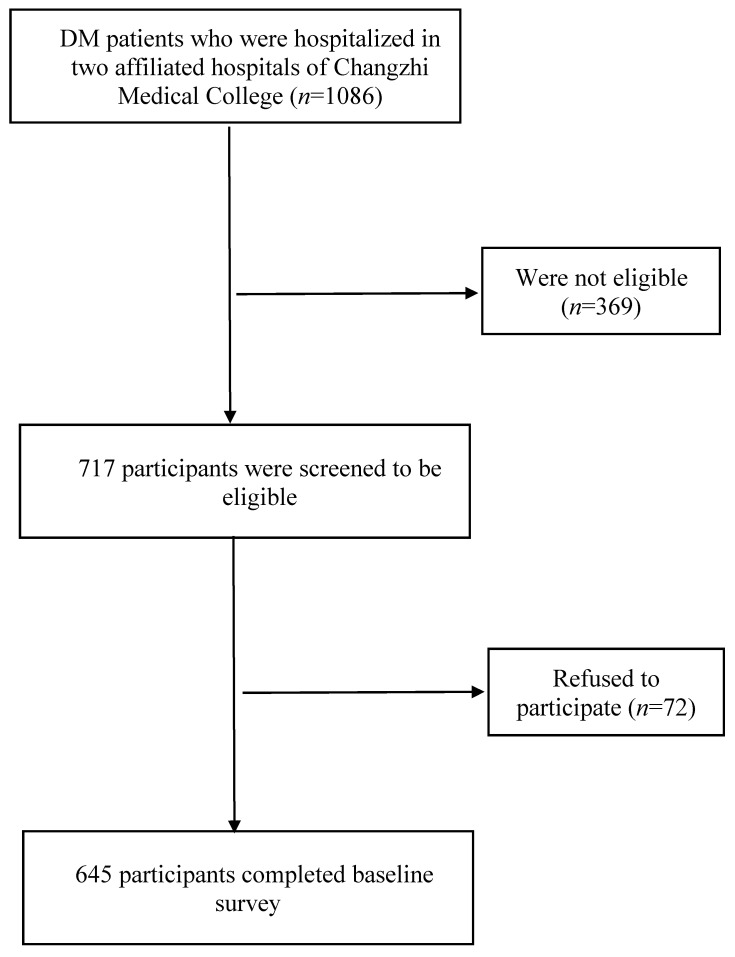
Flowchart of data collection.

The participating hospitals’ medical staff approached all inpatients diagnosed with DM in the endocrinology departments, screened their eligibility, briefed them about the study, and invited them to complete a face-to-face interview. The medical staff informed prospective eligible participants that their responses would be anonymous. They had the right to withdraw from the study, and refusal to participate would not affect their access to any services. Written informed consent was sought. Among 717 eligible inpatients with DM, 72 refused to participate, and 645 provided written informed consent and completed the face-to-face interview, as shown in Figure 1. No incentives were delivered to the participants. This study was conducted following the guidelines of the Declaration of Helsinki, and ethics approval was obtained by the Institutional Review Board of Changzhi Medical College (reference: 2021065).

### 2.4. Measures

#### 2.4.1. Development of the Questionnaire

A panel of epidemiologists, statisticians, and endocrinologists developed and finalized the questionnaire before the study implementation. 

#### 2.4.2. Background Characteristics

Sociodemographic information was collected, including gender, age, ethnicity, highest educational level, relationship status, employment status, monthly personal income, types of medical insurance, and lifestyles. The medical staff extracted characteristics of DM and other chronic diseases from participants’ medical records, such as type of DM, family history of DM, presence of chronic DM complications, glycemic control, and diagnosis of other chronic diseases that were not considered as chronic DM complications.

#### 2.4.3. COVID-19 Vaccination Uptake

The number of doses of COVID-19 vaccination taken up by the participants was extracted from their vaccination records. This study used uptake of at least one dose of COVID-19 vaccination as the dependent variable. A checklist was used to assess local adverse events (pain, redness, itching, swelling, induration, and skin rashes in the arm where the shot was given) and systematic adverse events (fatigue, malaise, headache, dizziness, lethargy, joint or muscle pain, fever, nausea, vomiting, diarrhea, and others) within one month of receiving COVID-19 vaccination. The same checklist was used to measure self-reported adverse events following COVID-19 vaccination among the general population in China [28].

#### 2.4.4. Presence of Chronic DM Complications

We defined chronic DM complications such as neuropathy, nephropathy, eye damage, cardiovascular and cerebrovascular diseases, coronary heart diseases, stroke, and peripheral vascular diseases. Previous studies commonly used the same definition [9]. Medical staff extracted the presence of specific types of chronic DM complications from participants’ medical records. 

#### 2.4.5. Perceptions Related to COVID-19 Vaccination Based on the HBM

Participants responded to the following items based on the HBM (response categories: 1 = strongly disagree, 2 = disagree, 3 = neutral, 4 = agree, and 5 = strongly agree). These items included: (1) one item measuring perceived susceptibility to COVID-19 (“You have a high risk of contracting COVID-19”); (2) one item measuring perceived severity of COVID-19 (“The consequences of contracting COVID-19 is severe”); (3) three items assessing perceived benefits of COVID-19 vaccination (“Receiving COVID-19 vaccination could reduce your risk of contracting COVID-19”, “Receiving COVID-19 vaccination could reduce your risk of transmitting COVID-19 to others” and “Receiving COVID-19 vaccination is beneficial for you and others”); (4) two items measuring perceived barriers (“You worried about the safety of COVID-19 vaccination for DM patients” and “You concern about the side effects of COVID-19 vaccination”); and (5) three items assessing cues to action (“Doctors will suggest you to receive COVID-19 vaccination to reduce the risk of infection”, “Mass media suggest DM patients to receive COVID-19 vaccination”, and “Uptake of COVID-19 vaccination among your relatives would influence your decision to receive the vaccination”). These items were adapted from a published study targeting people with DM in China [22].

### 2.5. Sample Size Planning

Our target sample size was 600 DM inpatients. We assumed that 50% of the DM inpatients had at least one chronic DM complication. Given a statistical power of 0.8 and an alpha value of 0.05, the sample size of the subgroup of participants with chronic DM complications (*n* = 300) and the subgroup without any complications (*n* = 300) could detect the smallest between-group difference of 7.9% in COVID-19 vaccination uptake. 

### 2.6. Statistical Analysis

The differences in background characteristics, COVID-19 vaccination uptake levels, and perceptions related to COVID-19 vaccination between people with at least one chronic DM complication and those without any such complications were compared using linear regression (for continuous variables) or Chi-square tests (for categorical variables). We compared the prevalence of specific types and any types of self-reported adverse events among vaccinated participants with and without chronic DM complications. Using uptake of at least one dose of COVID-19 vaccination as the dependent variable, this study investigated factors associated with the dependent variable in sub-samples of people with chronic DM complications and those without such complications. Associations between background characteristics and the dependent variable were first investigated using univariate logistic regression models, and crude odds ratios (ORs) were obtained. After adjusting for background characteristics with *p* < 0.05 in univariate analysis, the associations between perceptions based on the HBM and the dependent variable were then obtained by adjusted odds ratios (AORs). Each AOR was obtained by fitting a single logistic regression model involving one of the independent variables of interest and all significant background characteristics. SPSS 26.0 (Chicago, IL, USA) was used for data analysis, and *p* < 0.05 was considered statistically significant.

## 3. Results

### 3.1. Background Characteristics

The majority of participants were Han Chinese (98.1%), married or cohabiting with a partner (88.2%), and without full-time work (72.2%). Over half of them were male (56%), aged 50–69 years (54.3%), and had not attended secondary schools (58.8%). Fewer than half of them had a monthly personal income of less than CNY 2000 (40.8%) or had urban workers’ medical insurance (46.0%). Regarding lifestyles and health status, 95% were type 2 DM, 61.6% and 64.7% were current smokers and current drinkers, respectively, 56.4% reported a family history of DM, and 31% received a first DM diagnosis more than 10 years previously. The prevalence of chronic DM complications was 36.9% for eye damage, 3.7% for foot damage, 7.4% for neuropathy, and 15.2% for nephropathy. Among all participants, 365 (56.6%) had at least one chronic DM complication. Among participants with chronic DM complications, 328 had only one complication and 37 had at least two complications. 

When comparing participants with chronic DM complications (*n* = 365) and without complications (*n* = 280), significant differences were found in the following: educational level, relationship status, type of medical insurance, family history of DM, time since DM diagnosis, postprandial blood glucose level, and presence of chronic diseases that were not considered as chronic DM complications. (Table 1). 

### 3.2. Comparing COVID-19 Vaccination Uptake and Perceptions Related to COVID-19 Vaccination between DM Patients with and without Complications

**Figure 2:**
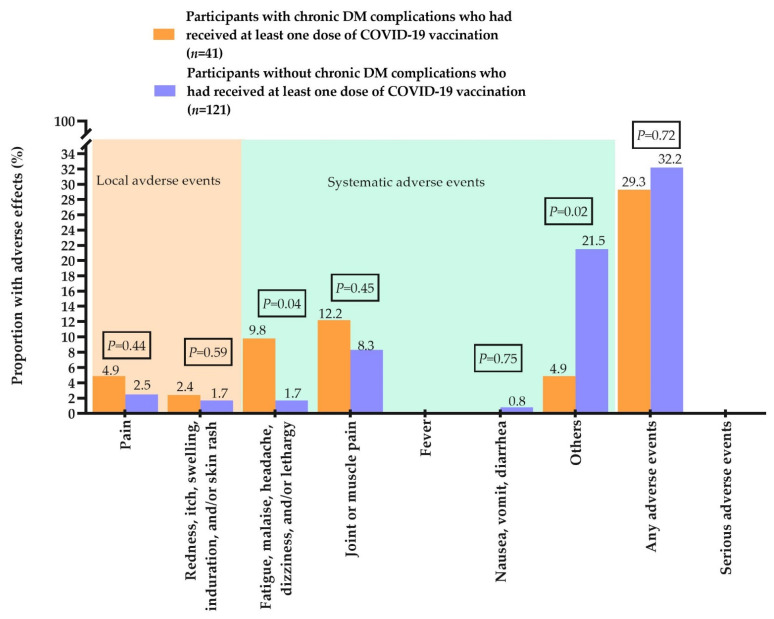
Adverse effects after vaccination.

As compared to participants with chronic DM complications, those without any complications reported significantly higher uptake of at least one dose of COVID-19 vaccination (43.2% versus 11.2%, *p* < 0.001) (Table 2). There was no between-group difference in the prevalence of any adverse events of COVID-19 vaccination (without complications: 32.2%, versus with complications: 29.3%, *p* = 0.72), and no serious adverse events were reported during the study (Figure 2 and Appendix A). 

Regarding perceptions related to COVID-19 vaccination, participants without any chronic DM complications had higher levels of perceived susceptibility, perceived severity, perceived benefits, and cues to action when compared to their counterparts with such complications. In contrast, participants without any chronic DM complications were less concerned about the safety of COVID-19 vaccination when compared to participants with such complications (Table 2). 

### 3.3. Factors Associated with COVID-19 Vaccination Uptake among Participants with Chronic DM Complications 

In univariate analysis, being of older age, having urban residents’ medical insurance or participation in the New Rural Cooperative Medical Scheme, and a longer time since DM diagnosis were all associated with higher COVID-19 vaccination uptake. Being a current smoker or drinker, having a family history of DM, and the presence of chronic conditions that were not considered as chronic DM complications were all negatively associated with the dependent variable (Table 3). 

After adjustment for these significant background characteristics, a perception of higher risk (AOR: 2.01, 95%CI: 1.03, 3.91) and of severer consequences of COVID-19 infection (AOR: 1.73, 95%CI: 1.17, 2.56), a belief that doctors would suggest that they receive COVID-19 vaccination (AOR: 1.99, 95%CI: 1.19, 3.35), and a belief that relatives’ vaccination uptake would influence their own decision to receive a COVID-19 vaccination (AOR: 2.80, 95%CI: 1.83, 4.30) were all associated with higher COVID-19 vaccination uptake. Concerns about the safety of COVID-19 vaccination (AOR: 0.37, 95%CI: 0.22, 0.62) and the side effects of vaccination (AOR: 0.50, 95%CI: 0.31, 0.83) for DM patients were negatively associated with the dependent variable (Table 4). 

### 3.4. Factors Associated with COVID-19 Vaccination Uptake among Participants without any Chronic DM Complications

In univariate analysis, having a tertiary or above educational level and a monthly personal income of more than CNY 3500 were associated with higher COVID-19 vaccination uptake. Being female, being a current smoker or drinker, being of older age, participation in the New Rural Cooperative Medical Scheme, having a family history of DM, and the presence of chronic conditions that were not considered as chronic DM complications were all negatively associated with the dependent variable (Table 3). 

After adjustment for these significant background characteristics, perceived severer consequences of COVID-19 infection (AOR: 2.74, 95%CI: 1.68, 4.47), a belief that receiving COVID-19 vaccination could reduce the risk of infection (AOR: 1.91, 95%CI: 1.28, 2.84), and a belief that relatives’ vaccination uptake would influence their decision to receive a COVID-19 vaccination (AOR: 2.09, 95%CI: 1.47, 2.96) were associated with higher COVID-19 vaccination uptake. Concerns about the safety of COVID-19 vaccination (AOR: 0.31, 95%CI: 0.18, 0.52) and the side effects of vaccination for DM patients (AOR: 0.58, 95%CI: 0.38, 0.89) were negatively associated with the dependent variable. (Table 4).

## 4. Discussion

To the best of our knowledge, this is one of the first studies comparing COVID-19 vaccination uptake and its associated factors between DM patients with and without chronic complications in China. This study showed that prevalence of COVID-19 vaccination uptake, levels of perceptions related to COVID-19 vaccination, and factors associated with COVID-19 vaccination uptake were different among participants with and with DM chronic complications. Such information provides a knowledge basis to develop tailored strategies to increase the vaccination coverage in these groups.

Our study found that the prevalence of COVID-19 vaccination uptake in all people with DM (25.1%) was significantly lower than that reported among the general population (88%) in China during the same period [29]. COVID-19 vaccination uptake was especially low among those with chronic DM complications (11.2%). The uptake rate among all participants was much lower than that observed among people with DM in other countries (e.g., 84.8% of people with DM in Saudi Arabia received at least one dose of COVID-19 vaccination) [25]. At the time of study, China was hit by the pandemic caused by Omicron sub-variant BA.2 of COVID-19. People with DM, especially those with chronic DM complications, are more vulnerable to severe consequences of Omicron sub-variant BA.2. Low levels of vaccination coverage therefore raised severe concerns and highlighted an urgent need to increase COVID-19 vaccination among people with DM in China, with a special focus on those with chronic DM complications. 

In contrast to our hypothesis, people with chronic DM complications had lower perceived susceptibility and severity of COVID-19 than those without such complications. They might not have been aware of the association between the presence of chronic complications and the risk and severity of COVID-19, as COVID-19 vaccination promotion materials seldom mention such information. In line with our hypothesis, people with chronic DM complications had more concerns about the efficacy and safety of COVID-19 vaccination, as fewer of them perceived such vaccination was beneficial for them, and more of them had doubts about its safety. Such differences can partially be explained by the lack of evidence comparing the efficacy and side effects of COVID-19 vaccination between DM patients with and without chronic complications. In addition, we found that people with chronic DM complications are less influenced by their relatives than those without any complications. The behaviors of family members are unlikely to dispel concerns about vaccine efficacy and safety in this group. 

The findings provided some empirical implications for developing health promotion programs tailored to DM patients with and without chronic complications. For both groups, future programs should give more attention to people with DM who are current smokers and drinkers, who have a family history of DM, and who have other chronic conditions as these people reported lower COVID-19 vaccination uptake. For people with chronic DM complications, additional efforts should target younger individuals, those with urban workers’ medical insurance and those with a shorter time since diagnosis. For people without such complications, future programs should focus on females, older individuals, those with lower socioeconomic status and those who are part of the New Rural Cooperative Medical Scheme. 

Different strategies for promoting COVID-19 vaccination should be applied to DM patients with and without chronic complications. First, the fear appeal approach, which has been widely used to promote recommended preventive behaviors [30,31], may be applicable for COVID-19 vaccination promotion among people with chronic DM complications. In our study, perceived susceptibility and perceived severity of COVID-19 infection were both significantly associated with higher COVID-19 vaccination uptake. Such individuals may perceive COVID-19 as a threat and hence be motivated to receive the vaccination against COVID-19. Based on the Extended Parallel Process model [32], only the presence of both perceived susceptibility and perceived severity can generate a perceived threat sufficient to motivate the processing of further messages about COVID-19. However, such an approach may not be appropriate for participants without any complications, as perceived susceptibility was not significantly associated with COVID-19 vaccination uptake in this group. Second, emphasizing the benefits of COVID-19 vaccination, which was commonly used and effectively promoted COVID-19 vaccination [33], may be useful for people with DM without any complications. However, such an approach might not be applicable for people with chronic DM complications as the association between perceived benefits and COVID-19 vaccination uptake in this group was not statistically significant. Moreover, physicians’ recommendations might effectively promote COVID-19 vaccination among people with chronic conditions. A previous study suggested that people with chronic DM complications were more willing to follow physicians’ suggestions, as they had experienced a longer hospital stay when compared to their counterparts who do not have such complications [34]. However, such recommendations given by physicians might not be useful in motivating DM patients without chronic DM complications to receive COVID-19 vaccination. 

In addition, some similar strategies can be applied to both groups for promoting COVID-19 vaccination uptake. First, it is important to remove concerns about the safety and side effects of COVID-19 vaccination, as these concerns were associated with lower COVID-19 vaccination uptake in both groups. Health communication messages should emphasize that COVID-19 vaccination is safe for all people with DM and that most have only mild side effects after vaccination. Positive testimonials shared by vaccinated people with DM may alleviate their concerns about COVID-19 vaccination and enhance vaccination coverage. Second, family members or relatives’ COVID-19 vaccination uptake positively influenced COVID-19 vaccination uptake in both groups of DM patients. Future programs should involve family members of people with DM. They might serve as role models and convince people with DM to receive COVID-19 vaccination. 

This study had some limitations. First, we only targeted inpatients, so our findings could not be generalized to all people with DM. People with DM who do not stay in hospitals might have better control of their DM and fewer chronic complications, and they may have higher COVID-19 vaccination uptake than inpatients. Second, the sample was not representative of all people with DM in China because it was limited to one city. Third, selection bias might exist, as we could not obtain characteristics of refusals. These individuals might have had different characteristics as compared to participants. Moreover, we did not include perceived self-efficacy in the measurement, as self-efficacy is an important determinant of vaccination uptake. Last but not least, the causal relationship between predictors and outcome variables could not be determined because the study was a cross-sectional study.

## 5. Conclusions

In conclusion, we found that people with DM reported a relatively low uptake of COVID-19 vaccination. Uptake rate was especially low among people with chronic DM complications. Different strategies might promote COVID-19 vaccination uptake in DM patients with and without chronic complications. Using the fear appeal approach and the recommendations of physicians are likely to succeed in people with chronic complications, while emphasizing the benefits of COVID-19 vaccination might work better among people without such complications. Some strategies, such as removing concerns about safety and side effects and using the influence of family members and relatives, are also useful to increase COVID-19 vaccination coverage in both groups. 

## Figures and Tables

**Table 1:** Comparing background characteristics between diabetes mellitus (DM) patients with and without complications.

	All (*n* = 645)	With Complications (*n* = 365)	Without Complications (*n* = 280)	*p* Values
	*n* (%)	*n* (%)	*n* (%)	
Sociodemographic				
Gender				
Male	361 (56.0)	212 (58.1)	149 (53.2)	0.22
Female	284 (44.0)	153 (41.9)	131 (46.8)	
Age group, years				
18–39	68 (10.5)	39 (10.7)	29 (10.4)	0.72
40–49	115 (17.8)	65 (17.8)	50 (17.9)	
50–59	187 (29.0)	113 (31.0)	74 (26.4)	
60–69	163 (25.3)	89 (24.4)	74 (26.4)	
≥70	112 (17.4)	59 (16.1)	53 (18.9)	
Ethnicity				
Han majority	633 (98.1)	357 (97.8)	276 (98.6)	0.48
Other ethnic minorities	12 (1.9)	8 (2.2)	4 (1.4)	
The highest educational level attained				
Primary	379 (58.8)	198 (54.2)	181 (64.6)	0.008
Secondary	122 (18.9)	70 (19.2)	52 (18.6)	
Tertiary or above	144 (22.3)	97 (26.6)	47 (16.8)	
Relationship status				
Currently single	76 (11.8)	35 (9.6)	41 (14.6)	0.048
Married or cohabiting with a partner	569 (88.2)	330 (90.4)	239 (85.4)	
Employment status				
Fulltime	179 (27.8)	100 (27.4)	79 (28.2)	0.82
Part-time/unemployed /retired/students	466 (72.2)	265 (72.6)	201 (71.8)	
Monthly personal income, China Yuan (USD)				
<2000 (317.5)	263 (40.8)	147 (40.3)	116 (41.4)	0.83
2000–3499 (317.5–555.4)	178 (27.6)	100 (27.4)	78 (27.9)	
3500–4999 (555.5–793.5)	131 (20.3)	73 (20.0)	58 (20.7)	
≥5000 (793.6)	73 (11.3)	45 (12.3)	28 (10.0)	
Type of medical insurance				
Urban workers’ medical insurance	297 (46.0)	202 (55.3)	95 (33.9)	<0.001
Urban residents’ medical insurance	152 (23.6)	115 (31.5)	37 (13.2)	
New Rural Cooperative Medical Scheme	196 (30.4)	48 (13.2)	148 (52.9)	
Lifestyles				
Current smoker				
No	248 (38.4)	134 (36.7)	114 (40.7)	0.30
Yes	397 (61.6)	231 (63.3)	166 (59.3)	
Current drinker				
No	228 (35.3)	118 (32.3)	110 (39.3)	0.07
Yes	417 (64.7)	247 (67.7)	170 (60.7)	
Characteristics related to DM				
Type of DM				
Type 2	613 (95.0)	344 (94.2)	269 (96.1)	
Type 1	32 (5.0)	21 (5.8)	11 (3.9)	0.29
Family history of DM				
No	281 (43.6)	146 (40.0)	135 (48.2)	0.04
Yes	364 (56.4)	219 (60.0)	145 (51.8)	
Time since receiving the diagnosis of DM, years				
≤1	180 (27.9)	99 (27.2)	81 (28.9)	0.002
2–10	265 (41.1)	133 (36.4)	132 (47.1)	
>10	200 (31.0)	133 (36.4)	67 (24.0)	
Fasting blood glucose level in the most recent episode of testing, mmol/L				
<7	236 (36.6)	134 (36.7)	102 (36.4)	0.37
7–13.9	367 (56.9)	203 (55.6)	164 (58.6)	
>13.9	42 (6.5)	28 (7.7)	14 (5.0)	
The postprandial blood glucose level in the most recent episode of testing, mmol/L				
<10	221 (34.3)	118 (32.3)	103 (36.8)	0.01
10–11.1	117 (18.1)	56 (15.3)	61 (21.8)	
>11.1	307 (47.6)	191 (52.4)	116 (41.4)	
Presence of chronic conditions that were not considered as chronic DM complications				
No	162 (25.1)	33 (9.0)	129 (46.1)	<0.001
Yes	483 (74.9)	332 (91.0)	151 (53.9)	

**Table 2:** Comparing COVID-19 vaccination uptake and perceptions related to COVID-19 vaccination between diabetes mellitus (DM) patients with and without complications.

	With Complications (*n* = 365)	Without Complications (*n* = 280)	Unadjusted *p* Values ^1^	Adjusted *p* Values ^2^
COVID-19 vaccination uptake				
Uptake of at least one dose of COVID-19 vaccination, *n* (%)				
No	324 (88.8)	159 (56.8)		
Yes	41 (11.2)	121 (43.2)	<0.001	<0.001
Perceived susceptibility				
You have a high risk of contracting COVID-19, *n* (%) agree/strongly agree	277 (75.9)	237 (84.6)	0.006	0.001
Item score, mean (SD)	3.8 (0.8)	4.1 (0.9)	<0.001	<0.001
Perceived severity				
The consequences of contracting COVID-19 are severe; *n* (%) agree/strongly agree	127 (34.8)	127 (45.3)	0.007	0.04
Item score, mean (SD)	2.9 (1.3)	3.5 (0.7)	<0.001	<0.001
Perceived benefits				
Receiving COVID-19 vaccination could reduce your risk of contracting COVID-19; *n* (%) agree/strongly agree	131 (35.9)	141 (50.4)	<0.001	0.03
Item score, mean (SD)	3.3 (0.8)	3.6 (0.8)	<0.001	<0.001
Receiving COVID-19 vaccination could reduce your risk of transmitting COVID-19 to others; *n* (%) agree/strongly agree	149 (40.8)	96 (34.3)	0.09	0.50
Item score, mean (SD)	3.3 (0.9)	3.3 (0.7)	0.43	0.71
Receiving COVID-19 vaccination is beneficial for you and others; *n* (%) agree/strongly agree	20 (5.5)	37 (13.2)	0.001	0.01
Item score, mean (SD)	2.8 (0.6)	3.0 (0.7)	0.001	0.006
Perceived barriers				
You are worried about the safety of COVID-19 vaccination for DM patients, *n* (%) agree/strongly agree	241 (66.0)	154 (55.0)	0.004	0.22
Item score, mean (SD)	3.8 (0.9)	3.5 (0.7)	<0.001	0.002
You are concerned about the side effects of COVID-19 vaccination; *n* (%) agree/strongly agree	186 (51.0)	124 (44.3)	0.09	0.28
Item score, mean (SD)	3.5 (0.8)	3.6 (0.9)	0.06	0.10
Cues to action				
Doctors will suggest you receive COVID-19 vaccination to reduce the risk of infection; *n* (%) agree/strongly agree	233 (63.8)	171 (61.1)	0.47	0.99
Item score, mean (SD)	3.5 (1.1)	3.6 (0.7)	0.10	0.10
Mass media suggest DM patients receive COVID-19 vaccination; *n*) agree/strongly agree	172 (47.1)	152 (54.3)	0.07	0.07
Item score, mean (SD)	3.2 (1.1)	3.5 (0.7)	<0.001	0.001
Uptake of COVID-19 vaccination among your relatives would influence your decision to receive the vaccine; *n* (%) agree/strongly agree	79 (21.6)	94 (33.6)	0.001	0.001
Item score, mean (SD)	2.3 (1.1)	3.2 (0.9)	<0.001	<0.001

^1^ Unadjusted *p* values: *p* values obtained by using univariate linear regression (for continuous variables) or Chi-square tests (for categorical variables) ^2^ Adjusted *p* values: *p* values adjusted for background characteristics with significant between-group difference (highest education level attained, relationship status, type of medical insurance, family history of DM, time since diagnosis of DM, postprandial blood glucose level in the most recent episode of testing, and presence of chronic conditions that were not considered as chronic DM complications). Adjusted *p* values were obtained using multivariate linear regression models (for continuous variables) or logistic regression models (for binary variables).

**Table 3:** Comparing COVID-19 vaccination uptake and perceptions related to COVID-19 vaccination between diabetes mellitus (DM) patients with and without complications.

	With Complications (*n* = 365)		Without Complications (*n* = 280)	
	OR (95%CI)	*p* Values	OR (95%CI)	*p* Values
Sociodemographic				
Gender				
Male	1.0		1.0	
Female	0.54 (0.27, 1.09)	0.09	0.39 (0.24, 0.64)	<0.001
Age group, years				
18–39	1.0		1.0	
40–49	4.57 (0.54, 38.78)	0.16	1.06 (0.42, 2.69)	0.90
50–59	4.10 (0.51, 32.85)	0.18	0.57 (0.24, 1.36)	0.20
60–69	4.28 (0.52, 34.97)	0.18	0.30 (0.12, 0.73)	0.008
≥70	10.74 (1.34, 85.86)	0.03	0.39 (0.16, 1.00)	0.049
Ethnicity				
Han majority	1.0		1.0	
Other ethnic minorities	1.13 (0.14, 9.44)	0.91	4.02 (0.41, 39.10)	0.23
The highest educational level attained				
Primary	1.0		1.0	
Secondary	0.53 (0.20, 1.45)	0.22	1.28 (0.68, 2.39)	0.44
Tertiary or above	0.89 (0.42, 1.88)	0.75	4.11 (2.05, 8.23)	<0.001
Relationship status				
Currently single	1.0		1.0	
Married or cohabiting with a partner	2.21 (0.51, 9.58)	0.29	1.38 (0.70, 2.74)	0.36
Employment status				
Full-time	1.0		1.0	
Part-time/unemployed /retired/students	1.96 (0.84, 4.57)	0.12	0.61 (0.36, 1.04)	0.07
Monthly personal income, China Yuan (USD)				
<2000 (317.5)	1.0		1.0	
2000–3499 (317.5–555.4)	0.43 (0.18, 1.04)	0.06	1.32 (0.72, 2.41)	0.37
3500–4999 (555.5–793.5)	0.42 (0.15, 1.15)	0.09	2.93 (1.53, 5.63)	0.001
≥5000 (793.6)	1.05 (0.42, 2.64)	0.92	10.22 (3.60, 29.04)	<0.001
Type of medical insurance				
Urban workers’ medical insurance	1.0		1.0	
Urban residents’ medical insurance	2.17 (1.02, 4.63)	0.05	1.20 (0.55, 2.61)	0.65
New Rural Cooperative Medical Scheme	3.99 (1.68, 9.48)	0.002	0.30 (0.17, 0.51)	<0.001
Lifestyles				
Current smoker				
No	1.0		1.0	
Yes	0.15 (0.07, 0.32)	<0.001	0.24 (0.15, 0.41)	<0.001
Current drinker				
No	1.0		1.0	
Yes	0.16 (0.08, 0.32)	<0.001	0.14 (0.08, 0.24)	<0.001
Characteristics related to DM				
Type of DM				
Type 2	1.0		1.0	
Type 1	0.38 (0.05, 2.91)	0.35	2.38 (0.68, 8.32)	0.18
Family history of DM				
No	1.0		1.0	
Yes	0.23 (0.12, 0.48)	<0.001	0.29 (0.18, 0.48)	<0.001
Time since receiving the diagnosis of DM, years				
≤1	1.0		1.0	
2–10	5.25 (1.16, 23.85)	0.03	0.88 (0.50, 1.53)	0.64
>10	11.79 (2.73, 50.96)	0.001	0.85 (0.44, 1.64)	0.64
Fasting blood glucose level in the most recent episode of testing, mmol/L				
<7	1.0		1.0	
7–13.9	0.92 (0.48, 1.79)	0.81	0.72 (0.44, 1.18)	0.19
>13.9	N.A.	N.A.	0.42 (0.12, 1.41)	0.16
The postprandial blood glucose level in the most recent episode of testing, mmol/L				
<10	1.0		1.0	
10–11.1	1.15 (0.43, 3.07)	0.78	0.67 (0.35, 1.27)	0.22
>11.1	1.00 (0.48, 2.08)	1.00	0.81 (0.47, 1.38)	0.43
Presence of chronic conditions that were not considered as chronic DM complications				
No	1.0		1.0	
Yes	0.12 (0.05, 0.27)	<0.001	0.21 (0.13, 0.36)	<0.001

OR: crude odds ratio. CI: confidence interval.

**Table 4:** Associations between perceptions and COVID-19 vaccination uptake among diabetes mellitus (DM) patients with and without complications.

	With Complications (*n* = 365)		Without Complications (*n* = 280)	
	AOR (95%CI)	*p* Values	AOR (95%CI)	*p* Values
Perceived susceptibility				
You have a high risk of contracting COVID-19	2.01 (1.03, 3.91)	0.04	0.88 (0.60, 1.28)	0.50
Perceived severity				
The consequences of contracting COVID-19 are severe	1.73 (1.17, 2.56)	0.01	2.74 (1.68, 4.47)	<0.001
Perceived benefits				
Receiving COVID-19 vaccination could reduce your risk of contracting COVID-19	1.21 (0.75, 1.95)	0.45	1.91 (1.28, 2.84)	0.001
Receiving COVID-19 vaccination could reduce your risk of transmitting COVID-19 to others	1.06 (0.67, 1.69)	0.81	0.89 (0.57, 1.41)	0.62
Receiving COVID-19 vaccination is beneficial for you and others	0.80 (0.45, 1.42)	0.45	0.63 (0.38, 1.05)	0.08
Perceived barriers				
You are worried about the safety of COVID-19 vaccination for DM patients	0.37 (0.22, 0.62)	<0.001	0.31 (0.18, 0.52)	<0.001
You are concerned about the side effects of COVID-19 vaccination	0.50 (0.31, 0.83)	0.008	0.58 (0.38, 0.89)	0.01
Cues to action				
Doctors will suggest you receive COVID-19 vaccination to reduce the risk of infection	1.99 (1.19, 3.35)	0.009	1.38 (0.89, 2.16)	0.16
Mass media suggest DM patients receive COVID-19 vaccination	1.08 (0.73, 1.59)	0.71	1.09 (0.70, 1.69)	0.72
Uptake of COVID-19 vaccination among your relatives would influence your decision to receive the vaccination	2.80 (1.83, 4.30)	<0.001	2.09 (1.47, 2.96)	<0.001

AOR: adjusted odds ratio. Odds ratios adjusted for significant background characteristics listed in Table 3.

## Data Availability

The data presented in this study are available from the corresponding author upon request. The data are not publicly available as they contain sensitive personal behaviors.

## Appendix A

**Table A1:** Comparing Self-Reported Adverse Events between Participants with and without Chronic Diabetes Mellitus (DM) Complications Who Have Received COVID-19 Vaccination.

Self-Reported Adverse Events of COVID-19 Vaccination	With Complications (*n* = 41)	Without Complications (*n* = 121)	*p* Values
Local adverse events (% Yes)			
Pain	4.9	2.5	0.44
Redness, itch, swelling, induration, and/or skin rash	2.4	1.7	0.59
Systematic adverse events (% Yes)			
Fatigue, malaise, headache, dizziness, and/or lethargy	9.8	1.7	0.04
Joint or muscle pain	12.2	8.3	0.45
Fever	0.0	0.0	N.A.
Nausea, vomiting, diarrhea	0.0	0.8	0.75
Others	4.9	21.5	0.02
Any adverse events (% Yes)	29.3	32.2	0.72
Serious adverse events (% Yes)	0.0	0.0	N.A.

N.A.: not applicable.

## References

[1] GBD 2019 Diabetes Mortality Collaborators (2022). Diabetes mortality and trends before 25 years of age: An analysis of the Global Burden of Disease Study 2019. Lancet Diabetes Endocrinol..

[2] Ma R.C.W. (2018). Epidemiology of diabetes and diabetic complications in China. Diabetologia.

[3] Chee Y.J., Tan S.K., Yeoh E. (2020). Dissecting the interaction between COVID-19 and diabetes mellitus. J. Diabetes Investig..

[4] Aldossari K.K., Alharbi M.B., Alkahtani S.M., Alrowaily T.Z., Alshaikhi A.M., Twair A.A. (2021). COVID-19 vaccine hesitancy among patients with diabetes in Saudi Arabia. Diabetes Metab. Syndr..

[5] Kreuzer D., Nikoopour E., Au B.C., Krougly O., Lee-Chan E., Summers K.L., Haeryfar S.M.M., Singh B. (2015). Reduced interferon-α production by dendritic cells in type 1 diabetes does not impair immunity to influenza virus. Clin. Exp. Immunol..

[6] Muniyappa R., Gubbi S. (2020). COVID-19 pandemic, coronaviruses, and diabetes mellitus. Am. J. Physiol. Endocrinol. Metab..

[7] Huang C., Wang Y., Li X., Ren L., Zhao J., Hu Y., Zhang L., Fan G., Xu J., Gu X. (2020). Clinical features of patients infected with 2019 novel coronavirus in Wuhan, China. Lancet.

[8] Mao L., Jin H., Wang M., Hu Y., Chen S., He Q., Chang J., Hong C., Zhou Y., Wang D. (2020). Neurologic Manifestations of Hospitalized Patients With Coronavirus Disease 2019 in Wuhan, China. JAMA Neurol..

[9] Albulescu R., Dima S.O., Florea I.R., Lixandru D., Serban A.M., Aspritoiu V.M., Tanase C., Popescu I., Ferber S. (2020). COVID-19 and diabetes mellitus: Unraveling the hypotheses that worsen the prognosis (Review). Exp. Ther. Med..

[10] World Health Organization (2022). Episode #46—Diabetes & COVID-19. https://www.who.int/emergencies/diseases/novel-coronavirus-2019/media-resources/science-in-5/episode-46---diabetes-covid-19.

[11] Centers for Disease Control and Prevention The Advisory Committee on Immunization Practices’ Updated Interim Recommendation for Allocation of COVID-19 Vaccine—United States, December 2020. https://www.cdc.gov/mmwr/volumes/69/wr/mm695152e2.htm.

[12] European Centre for Disease Prevention and Control (2020). COVID-19 Vaccination and Prioritisation Strategies in the EU/EEA. https://www.ecdc.europa.eu/en/publications-data/covid-19-vaccination-and-prioritisation-strategies-eueea.

[13] American Diabetes Association (2022). What You Need to Know: Getting a COVID-19 Vaccine. https://www.diabetes.org/coronavirus-covid-19/vaccination-guide.

[14] National Health Commission of the People’s Republic of China (2021). The Guidelines for COVID-19 vaccination (first edition). http://www.nhc.gov.cn/xcs/yqfkdt/202103/c2febfd04fc5498f916b1be080905771.shtml.

[15] Liu Z., Fu C., Wang W., Xu B. (2010). Prevalence of chronic complications of type 2 diabetes mellitus in outpatients–A cross-sectional hospital based survey in urban China. Health Qual. Life Outcomes.

[16] Jia W., Gao X., Pang C., Hou X., Bao Y., Liu W., Wang W., Zuo Y., Gu H., Xiang K. (2009). Prevalence and risk factors of albuminuria and chronic kidney disease in Chinese population with type 2 diabetes and impaired glucose regulation: Shanghai diabetic complications study (SHDCS). Nephrol. Dial. Transplant. Off. Publ. Eur. Dial. Transpl. Assoc.—Eur. Ren. Assoc..

[17] McGurnaghan S.J., Weir A., Bishop J., Kennedy S., Blackbourn L.A.K., A McAllister D., Hutchinson S., Caparrotta T.M., Mellor J., Jeyam A. (2021). Risks of and risk factors for COVID-19 disease in people with diabetes: A cohort study of the total population of Scotland. Lancet Diabetes Endocrinol..

[18] Tamura R.E., Said S.M., de Freitas L.M., Rubio I.G.S. (2021). Outcome and death risk of diabetes patients with Covid-19 receiving pre-hospital and in-hospital metformin therapies. Diabetol. Metab. Syndr..

[19] PPal R., Bhadada S.K., Misra A. (2021). COVID-19 vaccination in patients with diabetes mellitus: Current concepts, uncertainties and challenges. Diabetes Metab. Syndr..

[20] Guaraldi F., Montalti M., Di Valerio Z., Mannucci E., Nreu B., Monami M., Gori D. (2021). Rate and Predictors of Hesitancy toward SARS-CoV-2 Vaccine among Type 2 Diabetic Patients: Results from an Italian Survey. Vaccines.

[21] Syed Alwi S.A.R., Rafidah E., Zurraini A., Juslina O., Brohi I.B., Lukas S. (2021). A survey on COVID-19 vaccine acceptance and concern among Malaysians. BMC Public Health.

[22] Wang Y., Duan L., Li M., Wang J., Yang J., Song C., Li J., Wang J., Jia J., Xu J. (2022). COVID-19 Vaccine Hesitancy and Associated Factors among Diabetes Patients: A Cross-Sectional Survey in Changzhi, Shanxi, China. Vaccines.

[23] Nachimuthu S., Viswanathan V. (2021). Trend in COVID-19 vaccination among people with diabetes: A short study from India. Diabetes Metab. Syndr..

[24] Duan L., Wang Y., Dong H., Song C., Zheng J., Li J., Li M., Wang J., Yang J., Xu J. (2022). The COVID-19 Vaccination Behavior and Correlates in Diabetic Patients: A Health Belief Model Theory-Based Cross-Sectional Study in China, 2021. Vaccines.

[25] Tourkmani A.M., Bin Rsheed A.M., AlEissa M.S., Alqahtani S.M., AlOtaibi A.F., Almujil M.S., Alkhashan I.H., Alnassar T.N., Alotaibi M.N., Alrasheedy A.A. (2022). Prevalence of COVID-19 Infection among Patients with Diabetes and Their Vaccination Coverage Status in Saudi Arabia: A Cross-Sectional Analysis from a Hospital-Based Diabetes Registry. Vaccines.

[26] Lee D., Rundle-Thiele S., Li G. (2021). Motivating Seasonal Influenza Vaccination and Cross-Promoting COVID-19 Vaccination: An Audience Segmentation Study among University Students. Vaccines.

[27] Carico R.R., Sheppard J., Thomas C.B. (2021). Community pharmacists and communication in the time of COVID-19: Applying the health belief model. Res. Soc. Adm. Pharm..

[28] Huang X., Yan Y., Su B., Xiao D., Yu M., Jin X., Duan J., Zhang X., Zheng S., Fang Y. (2022). Comparing Immune Responses to Inactivated Vaccines against SARS-CoV-2 between People Living with HIV and HIV-Negative Individuals: A Cross-Sectional Study in China. Viruses.

[29] (2022). Our World in Data. Coronavirus (COVID-19) Vaccinations. https://ourworldindata.org/covid-vaccinations?country=OWID_WRL.

[30] Lau J.T.F., Lee A.L., Tse W.S., Mo P.K.H., Fong F., Wang Z., Cameron L.D., Sheer V. (2016). A Randomized Control Trial for Evaluating Efficacies of Two Online Cognitive Interventions With and Without Fear-Appeal Imagery Approaches in Preventing Unprotected Anal Sex Among Chinese Men Who Have Sex with Men. AIDS Behav..

[31] Stolow J.A., Moses L.M., Lederer A.M., Carter R. (2020). How Fear Appeal Approaches in COVID-19 Health Communication May Be Harming the Global Community. Health Educ. Behav..

[32] Jahangiry L., Bakhtari F., Sohrabi Z., Reihani P., Samei S., Ponnet K., Montazeri A. (2021). Correction to: Risk perception related to COVID-19 among the Iranian general population: An application of the extended parallel process model. BMC Public Health.

[33] Ashworth M., Thunström L., Cherry T.L., Newbold S.C., Finnoff D.C. (2021). Emphasize personal health benefits to boost COVID-19 vaccination rates. Proc. Natl. Acad. Sci. USA.

[34] Cheng S.-W., Wang C.-Y., Ko Y. (2019). Costs and Length of Stay of Hospitalizations due to Diabetes-Related Complications. J. Diabetes Res..

